# Nrf2 Activation Is Involved in Cyclic Mechanical Stress-Stimulated Osteogenic Differentiation in Periodontal Ligament Stem Cells via PI3K/Akt Signaling and HO1-SOD2 Interaction

**DOI:** 10.3389/fcell.2021.816000

**Published:** 2022-01-06

**Authors:** Xun Xi, Zixuan Li, Hong Liu, Shuai Chen, Dongxu Liu

**Affiliations:** Department of Orthodontics, School and Hospital of Stomatology, Cheeloo College of Medicine, Shandong University & Shandong Key Laboratory of Oral Tissue Regeneration & Shandong Engineering Laboratory for Dental Materials and Oral Tissue Regeneration, Jinan, China

**Keywords:** nuclear factor erythroid-2-related factor-2, mechanical stress, orthodontic tooth movement, periodontal ligament, osteogenic differentiation

## Abstract

Nuclear factor erythroid-2-related factor-2 (Nrf2), the major transcriptional regulator in antioxidant response and cellular defense, had the vital effect on regulating osteogenic differentiation. Our previous study revealed that Nrf2 activation was involved in cyclic mechanical stress-stimulated osteogenic differentiation in the human periodontal ligament stem cells (PDLSCs). However, the mechanisms of Nrf2 underlying this process remained unclear. The goal of the study was to explore the mechanisms of Nrf2 in PDLSCs during cyclic mechanical stress-stimulated osteogenic differentiation via the tandem mass tag (TMT)-based liquid chromatography tandem-mass spectrometry (LC-MS/MS) analysis. And we applied tert-Butylhydroquinone (t-BHQ), the Nrf2 activator, to the orthodontic rats and detected the expression levels of the osteogenesis markers by immunohistochemistry (IHC) staining. Our results showed that Nrf2 activation in PDLSCs was involved in cyclic mechanical stress-stimulated osteogenic differentiation via phosphoinositide 3 kinase (PI3K)/protein kinase B (Akt) pathway. The protein-protein interaction between Akt and Nrf2 was detected. And the protein-protein interaction between heme oxygenase 1 (HO1) and superoxide dismutase 2 (SOD2), the downstream antioxidants of Nrf2, was associated with cyclic mechanical stress-stimulated osteogenic differentiation. T-BHQ enhanced the expression levels of the osteogenesis markers in orthodontic rats. Nrf2 might possess the potential to be a feasible molecular target in orthodontics.

## Introduction

The biological basis during orthodontic tooth movement is the alveolar bone remodeling. The alveolar bone remodeling is a continuous, complex and coordinated biological process, involving bone-forming mediated by osteoblasts and bone-resorbing mediated by osteoclasts in orthodontics. The mechanical stress-stimulated osteogenic differentiation in the human periodontal ligament stem cells (PDLSCs) is the vital component in alveolar bone remodeling in orthodontics at the tension side (Seo et al., 2004; Huang et al., 2018). However, some risks in orthodontic treatment, such as the loss of attachment, the alveolar bone loss, as well as the gingival recession, pose great challenges for orthodontists (Rafiuddin et al., 2015). And orthodontists are now exploring the molecular mechanisms and searching for the potential therapeutic molecular targets, in order to avoid the possible side effects and improve the alveolar bone remodeling during the orthodontic treatment.

Our previous study revealed that the cyclic mechanical stress enhanced the reactive oxygen species (ROS) level and induced the expression of nuclear factor erythroid-2-related factor-2 (Nrf2) and its downstream molecules, such as heme oxygenase 1 (HO1) and NADPH dehydrogenase quinone 1 (NQO1) during cyclic mechanical stress-stimulated osteogenic differentiation in PDLSCs (Xi et al., 2021). Tert-Butylhydroquinone (t-BHQ), the Nrf2 activator and the United States Food and Drug Administration (FDA)-approved food additive, increased the expression levels of osteogenic differentiation markers, including collagen type 1 (COL1), runt-related transcription factor 2 (RUNX2), osteopontin (OPN), and alkaline phosphatase (ALP) activity in PDLSCs during cyclic mechanical stress (Xi et al., 2021).

Nrf2, as the major transcriptional regulator, had the pivotal effect on the endogenous antioxidant response and cellular defense by regulating the expression and activity of antioxidant components, including HO1 and NQO1 (Dai et al., 2020). The previous studies supported the notion that Nrf2 was a vital factor in regulating osteoblast differentiation and bone formation (Sun et al., 2015; Wu et al., 2019). Nrf2 activation in osteoblast differentiation might be related to the intracellular ROS levels and the antioxidant components (Francisqueti-Ferron et al., 2019).

Accumulating studies showed that many complex mechanisms were involved in regulating the Nrf2 signaling at various levels, such as transcription, post-translational modification as well as protein-protein interactions. The complex mechanisms were associated with controlling the intracellular distribution, stability and activity of Nrf2 (Zhang et al., 2015). And many signaling pathways were vital for modulating Nrf2 activity, such as protein kinase C (PKC), phosphoinositide 3 kinase (PI3K)/protein kinase B (Akt) and p38 mitogen-activated protein kinase (MAPK) (Fão et al., 2019).

However, the precise molecular mechanisms of Nrf2 underlying cyclic mechanical stress-stimulated osteogenic differentiation are not fully elucidated. Better understanding of the mechanisms of Nrf2 underlying cyclic mechanical stress-stimulated osteogenic differentiation may make profound impacts on improving the alveolar bone remodeling in orthodontics.

Proteins are the critical executors in undertaking the life activities directly. It is especially important to examinate the proteins related to various physiological as well as pathological processes, in order to explore the intricate mechanisms in numerous biological processes. Along with the advances in proteomics technologies, it is feasible to find and identify the differentially expressed proteins to further study the potential, interrelated as well as multifactorial mechanisms (Cheng et al., 2021).

Our research was designed to explore the potential mechanisms of Nrf2 underlying cyclic mechanical stress-stimulated osteogenic differentiation via the tandem mass tag (TMT)-based liquid chromatography tandem-mass spectrometry (LC-MS/MS) analysis. And we applied t-BHQ, the Nrf2 activator, to the orthodontic rats. We detected the expression levels of the osteogenesis markers by immunohistochemistry (IHC) staining to confirm that Nrf2 might be a promising therapeutic target to improve the alveolar bone remodeling in orthodontics.

## Materials and Methods

### Culture and Identification of PDLSCs

This research was conducted in adherence with the Declaration of Helsinki. The ethical approval for this study was approved through the Medical Ethical Committee of the School of Stomatology, Shandong University (Protocol number: 20201206). The informed consent and the assent were taken from the participants as well as their guardians. The methods of the culture and identification of PDLSCs were in accordance with our previous research (Xi et al., 2021). The cultured PDLSCs were isolated from the human periodontal ligament (PDL) tissues in premolars from 12- to 16-year-old systemically healthy orthodontic individuals who undergone tooth extraction. 0.25% trypsin (Solarbio, Beijing, China) was applied to separate PDLSCs for passage. PDLSCs at passages 3–6 were utilized in the follow-up experiments.

The flow cytometric examinations of STRO-1, CD146, CD34, CD45 (Proteintech Group, Chicago, IL, United States) were carried out by using BD Accuri™ C6 flow cytometer (BD Biosciences, Milan, Italy) as described previously (Xi et al., 2021).

The multidirectional differentiation potential of PDLSCs was confirmed through alizarin red staining (Sigma-Aldrich; Merck KGaA, Darmstadt, Germany), oil red O staining (Solarbio) as well as alcian blue staining (Solarbio) as described previously (Xi et al., 2021).

### Cyclic Mechanical Stress

The application of cyclic mechanical stress (10% and 0.5 Hz) was carried out with Flexercell FX-5000 Strain Unit (Flexcell International Corporation, Hillsborough, NC, United States) as described previously (Xi et al., 2021). The PDLSCs were treated with 10% and 0.5 hz cyclic mechanical stress for 0 and 12 h. The PDLSCs in 0 h groups were the non-loaded cells without cyclic mechanical stress. Non-loaded PDLSCs (0 h) were cultured on the Bioflex ^®^ culture plates in the same incubator for the maximum tension time without cyclic mechanical stress.

### RNA Interference of Nrf2

The design and synthesis of the small interfering RNA (siRNA) was conducted by GenePharma Corporation (Shanghai, China). PDLSCs were transfected with Micropoly-transfecter TM cell reagent (Micropoly, Nantong, China). The transfection efficiency was detected and confirmed with qRT-PCR and western blotting as described previously (Xi et al., 2021). The siRNA sequences used in this experiment were listed below: siNrf2 (forward 5′-GGG​AGG​AGC​UAU​UAU​CCA​UTT-3′ and reverse 5′-AUG​GAU​AAU​AGC​UCC​UCC​CTT-3′); siSCR (forward 5′-UUC​UCC​GAA​CGU​GUC​ACG​UTT-3′ as well as reverse 5′-ACG​UGA​CAC​GUU​CGG​AGA​ATT-3′).

### TMT-Based LC-MS/MS Analysis

The samples were divided into two groups (the control group and siNrf2 group) and each group contained three repetitions. The samples were lysed by lysis buffer, including 7 M urea (Bio-Rad, Hercules, CA, United States), 2 M thiourea (Sigma-Aldrich), and 0.1% CHAPS (Bio-Rad). The supernatant of the sample was centrifuged at 14,000 g at 4°C for 30 min and then collected. The protein solution was incubated with the reducing reagent (5 μl 200 mM) (Thermo Scientific, Waltham, MA, United States) at 55°C for 1 h, iodoacetamide (5 μl 375 mM) (Thermo Scientific) at room temperature for 10 min, dissolution buffer (200 μl 100 mM) (Thermo Scientific) and then centrifuged at 12,000 g for 20 min. The trypsin (Thermo Scientific) was added overnight at room temperature.

The TMT reagent (Thermo Scientific) was used to label the peptides of the samples. The TMT reagent was dissolved in acetonitrile, added to the digested sample and incubated for 1 h at room temperature. The reaction was terminated through the addition of 5% hydroxylamine. The labeled samples were collected for LC-MS/MS analysis.

The tryptic peptide fractions were dissolved in buffer A (0.1% formic acid). The peptides were eluted at a flow rate of 600 nl/min with buffer B (80% acetonitrile and 0.1% formic acid). The Orbitrap Q Exactive HF-X mass spectrometer (Thermo Scientific) was used for LC-MS/MS analysis, using Nanospray Flex™ Ion Source (Thermo Scientific), with the spray voltage of 2.4 kV. The full MS scans were performed from 407 to 1,500 m/z at a resolution of 60,000. The automatic gain control (AGC) target was 3 × 10^6^, and the maximum ion injection time was 20 ms. For MS/MS scans, the 40 most abundant ions were selected for higher energy collisional dissociation fragmentation. The MS/MS scans were performed at a resolution of 45,000 (200 m/z), and AGC was 5 × 10^4^ and the maximum ion injection time was 86 ms. The collision energy was 32%. The data were analyzed by Proteome Discoverer 2.4 through *Homo sapiens* database. The parameters were set as follows. Static modification: carbamidomethyl (C); dynamic modification: M oxidation (15.995 Da), TMT-6 plex (K, N-terminal), acetyl (protein N-terminal); precursor ion mass tolerance: ±15 ppm; fragment ion mass tolerance: ±0.02 Da; max missed cleavages: 2. The proteins with the *p* value less than 0.05 and the fold change more than 1.2 or less than 0.833 were selected and recognized as differentially expressed proteins for further study.

The Gene Ontology (GO) analysis was carried out through UniProt GoA database (https://www.ebi.ac.uk/GOA/), and classified the proteins into three categories, including cellular components, molecular functions, and physiological processes. The pathway analyses were carried out by the Kyoto Encyclopedia of Genes and Genomes (KEGG) database (https://www.genome.jp/kegg/). The *p* value less than 0.05 was considered significant. The protein-protein interaction (PPI) information was analyzed by the STRING database (https://string-db.org/).

### Reagent Preparation

LY294002 (Cell Signaling Technology, Danvers, MA, United States), the PI3K inhibitor, was dissolved in dimethyl sulfoxide (DMSO), and the final concentration was 20 μM in PDLSCs (Chen et al., 2013; Kook et al., 2014; Jia et al., 2020).

The t-BHQ (Solarbio) solution was prepared in DMSO. The concentration of the t-BHQ solution was 10 μM in PDLSCs as described previously (Yoon et al., 2016; Xi et al., 2021).

### Quantitative Real-Time RT-PCR

The method of quantitative real-time RT-PCR was in accordance with our previous research (Xi et al., 2021). The primer sequences were showed in Supplementary Table S1.

### Western Blotting

The method of the experiment was in accordance with our previous research (Xi et al., 2021). COL1 (14695-1-AP, Proteintech), RUNX2 (ab23981, Abcam, Cambridge, MA, United States), OPN (22952-1-AP, Proteintech), Nrf2 (ab137550, Abcam), HO1 (10701-1-AP, Proteintech), superoxide dismutase 2 (SOD2) (24127-1-AP, Proteintech), Akt (10176-2-AP, Proteintech), p-Akt (66444-1-lg, Proteintech), GAPDH (sc-25778, Santa Cruz Biotechnology, Santa Cruz, CA, United States) and Lamin B1 (12987-1-AP, Proteintech) were used for western blotting.

### Co-Immunoprecipitation

PDLSCs were lysed with NP-40 (Beyotime, Nantong, China) on ice for 10 min, and the supernatant was collected after centrifugation. The supernatant samples were incubated with the primary antibodies (2.5 μg) and Protein A/G Plus Agarose (sc-2003, Santa) at 4°C overnight. And the primary antibodies used in this study for enriching the protein complex were presented below: Nrf2 (16396-1-AP, Proteintech), Akt (10176-2-AP, Proteintech), HO1 (10701-1-AP, Proteintech), SOD2 (24127-1-AP, Proteintech). After immunoprecipitation, the immunocomplexes were washed three times with NP-40 lysis buffer, collected after centrifugation, and then re-suspended with SDS-loading buffer, and boiled for 5 min for western blotting. The primary antibodies used for western blotting were presented below: Nrf2 (66504-1-lg, Proteintech), Akt (60203-2-lg, Proteintech), HO1 (66743-1-lg, Proteintech), SOD2 (66474-1-lg, Proteintech).

### Immunofluorescence Staining

The fixation of PDLSCs was performed with 4% paraformaldehyde for 15–20 min, and then incubated with 0.2% Triton X-100 (Solarbio) for 10 min for permeabilization. PDLSCs were blocked with 10% goat serum for 1 h at room temperature, and then incubated with anti-SOD2 primary antibody (1:200, 24127-1-AP, Proteintech), overnight at 4°C, and then incubated with CoraLite594-conjugated Goat Anti-Rabbit IgG (H+L) (SA00013-4, Proteintech) and protected from light for 1 h at room temperature. DAPI (Solarbio) staining for the nuclei in PDLSCs was carried out. PDLSCs were visualized using the fluorescence microscope (OLYMPUS, Tokyo, Japan).

### Experimental Animals

The ethical approval for the animal experiment was approved through the Medical Ethical Committee of School of Stomatology, Shandong University (Protocol number: 20201205). And the experiments were conducted in adherence with the National Institutes of Health Guide for the Care and Use of Laboratory Animals. The male Wistar rats aged 8 weeks were used in our experiment.

### Tooth Movement Models

The method was in accordance with our previous research (Xi et al., 2021). Mesial force of 25 g was carried out for mesial movement of the maxillary first molar with the maxillary incisors as the anchorage (An et al., 2017; Motoji et al., 2020).

Rats in this study were randomly allocated into the control group as well as t-BHQ group. 2 mg/ml t-BHQ solution in 1% DMSO in saline was injected intraperitoneally every second day. The rats in t-BHQ group were injected with t-BHQ solution (50 mg/kg) intraperitoneally (Lazaro et al., 2018; Xi et al., 2021). The control rats were injected with 1% DMSO in saline.

The rats were sacrificed at 7, 14 and 28 days after orthodontic treatment with an overdose of the pentobarbital anaesthesia, The cardiac perfusion with 4% paraformaldehyde was carried out to fix the tissue. Tissues were harvested and fixed using 4% paraformaldehyde.

### Immunohistochemistry Examinations

IHC examinations were carried out through the SPlink Detection kit (catalog no. SP9000, ZSGB-BIO, Beijing, China). Nrf2 (1:500, 16396-1-AP, Proteintech), HO1 (1:400, 10701-1-AP, Proteintech), ALP (1:400, 11187-1-AP, Proteintech) and COL1 (1:1,000, 14695-1-AP, Proteintech) were used for IHC examination. The region of PDL in the maxillary first molar around distal to the cervial third of mesiobuccal root was selected for detecting the protein levels (Nrf2, HO1, ALP as well as COL1) (Suzuki et al., 2016; An et al., 2017). The stained sections were visualized with the microscope (Olympus, Tokyo, Japan) and acquired by Olympus cellSens Standard 1.17 (Olympus). The Image-Pro Plus 6.0 software (Media Cybernetics, Silver Spring, MD, United States) was used to quantify the integrated optical density (IOD) of the protein levels (Nrf2, HO1, ALP as well as COL1).

### Statistical Analysis

Results were represented as mean ± standard deviation (SD). One-way ANOVA test and Student’s *t*-test were used as appropriate through the GraphPad Prism software (MacKiev Software, Boston, MA, United States).

## Results

### Culture and Identification of PDLSCs

**FIGURE 1:**
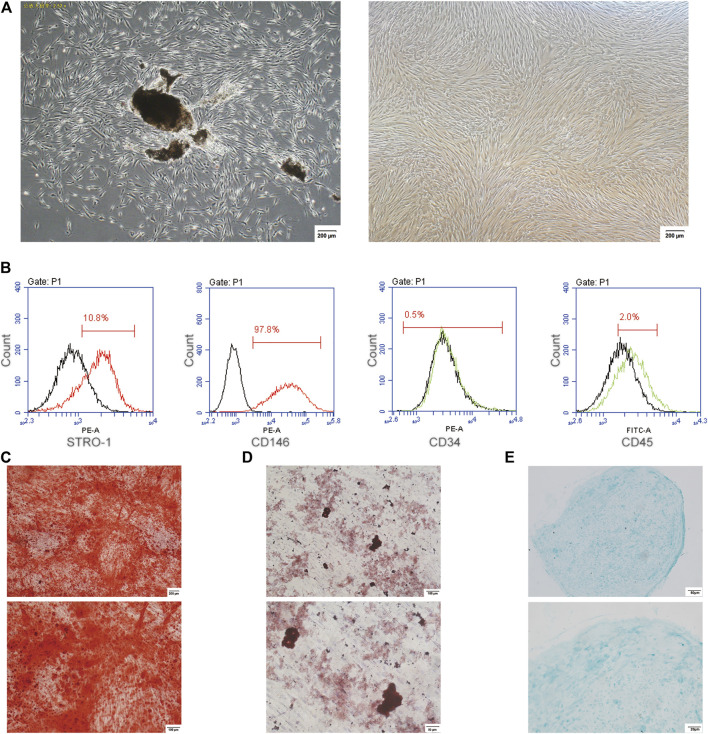
The culture and identification of PDLSCs. **(A)** The cultured PDLSCs, isolated from the human periodontal ligament tissues and passaged at 6, with a morphology of fusiform. The scale bars, 200 µm. **(B)** Flow cytometry data showed the positive expression for STRO-1 and CD146, and the negative expression for CD34 and CD45 in PDLSCs. **(C)** Alizarin red staining. The scale bars, 100 and 200 µm. **(D)** Oil red O staining. The scale bars, 50 and 100 µm. **(E)** Alcian blue staining. The scale bars, 20 and 50 µm.

The cultured PDLSCs, isolated from the human periodontal ligament tissues, showed the morphology of fusiform (Figure 1A). The flow cytometry data showed the positive expression for STRO-1 as well as CD146, and the negative expression for CD34 as well as CD45 in PDLSCs (Figure 1B). The cultured PDLSCs were cells with multidirectional differentiation potential, which was confirmed by alizarin red staining, oil red O staining as well as alcian blue staining (Figures 1C–E).

### Identification of the Differentially Expressed Proteins and Bioinformatics Through TMT-Based LC-MS/MS Analysis

**FIGURE 2:**
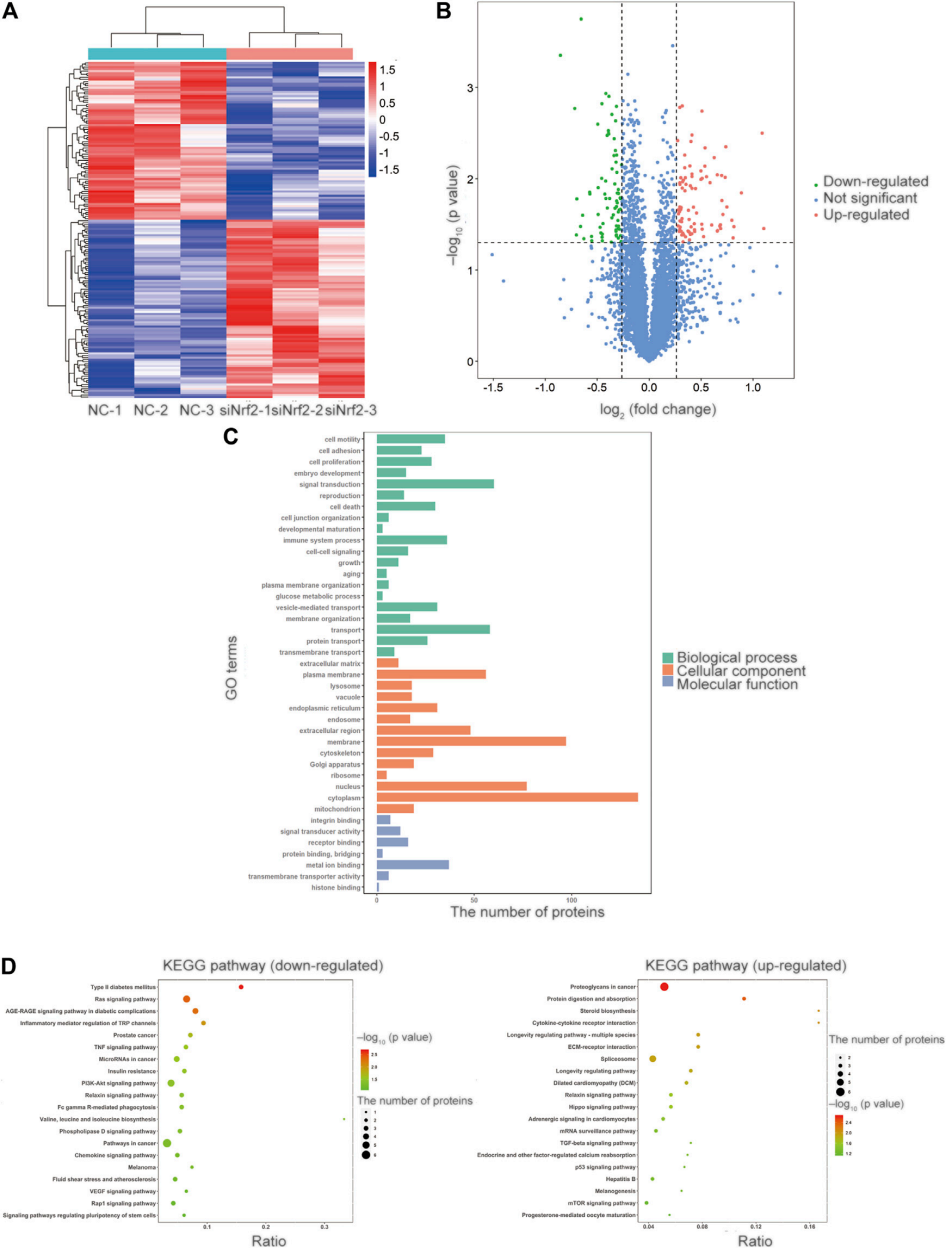
Identification of the differentially expressed proteins and bioinformatics via TMT-based LC-MS/MS analysis. **(A)** The heatmap of the relative abundance of proteins in each sample. **(B)** The volcano plot showed 162 proteins were differentially expressed compared with the control proteins, including 76 downregulated and 86 upregulated proteins. The proteins with the *p* value less than 0.05 and the fold change more than 1.2 or less than 0.833 were selected and recognized as differentially expressed proteins for further study. The value of log_2_ (fold change) was set as abscissa, and the value of -log_10_ (*p* value) was set as the ordinate. **(C)** GO enrichment analysis revealed that the differentially expressed proteins were involved in the cellular component (CC) category, the biological process (BP) category and the molecular function (MF) category. **(D)** The KEGG pathway enrichment analysis of the up-regulated and down-regulated proteins.

We investigated the potential molecular mechanisms of Nrf2 in cyclic mechanical stress-stimulated osteogenic differentiation in PDLSCs via LC-MS/MS. The results revealed that, compared with the controls, 162 proteins were differentially expressed, including 76 down-regulated and 86 up-regulated proteins (Figures 2A,B and Supplementary Table S2).

According to GO enrichment analysis, the differentially expressed proteins were mainly distributed in cytoplasm, membrane and nucleus in the cellular component (CC) category. We observed that the differentially expressed proteins were involved in signal transduction, transport and immune system process in the biological process (BP) category. And the differentially expressed proteins participated in metal ion binding, receptor binding and signal transducer activity in the molecular function (MF) category (Figure 2C).

In the KEGG pathway enrichment analysis of differentially expressed proteins (Figure 2D), PI3K/Akt signaling pathway attracted our attention, as its vital role in the activation and translocation of Nrf2 (Rai et al., 2019). PI3K/Akt signaling pathway was selected for further investigation in the subsequent study.

### The Involvement of PI3K/Akt Signaling Pathway in the Nrf2 Activation in PDLSCs During Cyclic Mechanical Stress-Stimulated Osteogenic Differentiation

**FIGURE 3:**
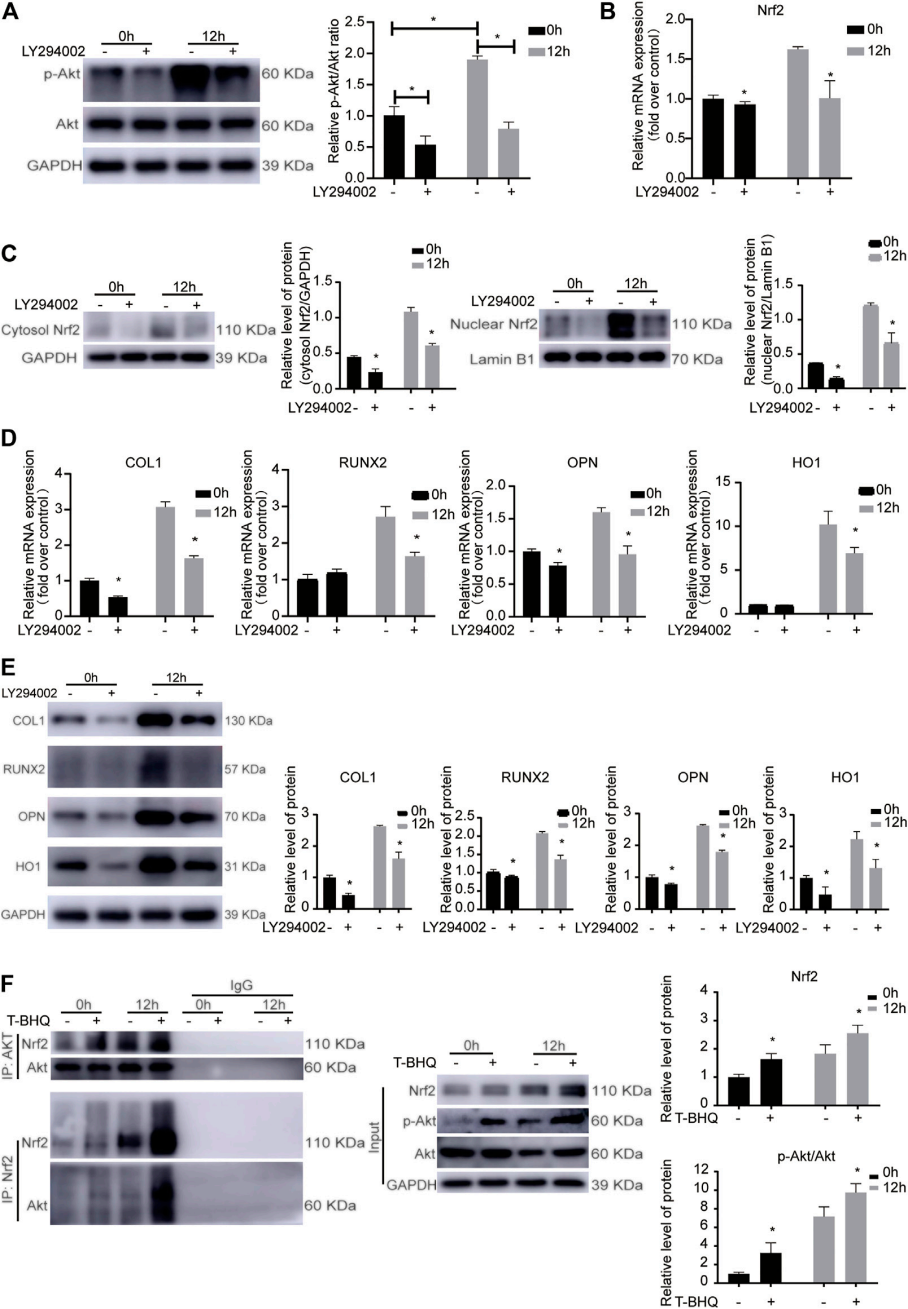
The involvement of PI3K/Akt signaling pathway in the Nrf2 activation during the cyclic mechanical stress-stimulated osteogenic differentiation of PDLSCs. **(A)** LY294002, the PI3K inhibitor, inhibited the level of p-Akt under cyclic mechanical stress based on the western blotting experiments. **(B)** LY294002 down-regulated the Nrf2 mRNA under cyclic mechanical stress in PDLSCs. **(C)** LY294002 decreased the cytosol and nuclear protein expression level of Nrf2 under cyclic mechanical stress in PDLSCs. **(D)** LY294002 inhibited the mRNA levels of osteogenic relative markers (COL1, RUNX2 and OPN) and HO1 under cyclic mechanical stress in PDLSCs. **(E)** LY294002 inhibited the protein expression levels of osteogenic relative markers (COL1, RUNX2 and OPN) and HO1 under cyclic mechanical stress in PDLSCs. **(F)** Co-IP experiments showed the protein-protein interaction between Akt and Nrf2 in PDLSCs as immunoprecipitation with anti-Akt antibody and anti-Nrf2 antibody to enrich the protein complex, respectively.

To confirm the involvement of PI3K/Akt signaling pathway in the Nrf2 activation in PDLSCs during the cyclic mechanical stress-stimulated osteogenic differentiation based on the KEGG pathway analysis, LY294002 was applied in the study. Cyclic mechanical stress increased the phosphorylation of Akt (p-Akt), while LY294002, the PI3K inhibitor, inhibited the level of p-Akt (Figure 3A and Supplementary Table S3). And LY294002 down-regulated the mRNA, the cytosol and nuclear protein expression level of Nrf2 (Figures 3B,C and Supplementary Table S3). The cyclic mechanical stress-stimulated osteogenic differentiation abilities with or without LY294002 in PDLSCs were measured. And the data showed that LY294002 inhibited the mRNA as well as the protein expression levels of osteogenic relative markers (COL1, RUNX2 and OPN) (Figures 3D,E and Supplementary Table S3). And the decreased mRNA and the decreased protein expression level of HO1 with LY294002 were observed. The abovementioned results showed that the PI3K/Akt pathway was associated with modulating the Nrf2 expression in cyclic mechanical stress-stimulated osteoblast differentiation of PDLSCs.

### The Protein-Protein Interaction Between Akt and Nrf2 in PDLSCs During the Cyclic Mechanical Stress-Stimulated Osteogenic Differentiation

To further confirm the involvement of PI3K/Akt signaling pathway in the Nrf2 activation in PDLSCs during the cyclic mechanical stress, in the subsequent study, we used the STRING database. And we found that the potential protein-protein interaction between Akt and Nrf2 might be associated with the process. Immunoprecipitation experiments were performed with anti-Nrf2 antibody to enrich the protein complex containing Nrf2, followed by western blotting with anti-Akt antibody. The results showed that Akt was present in the complex containing Nrf2 (Figure 3F). The similar results were observed when immunoprecipitation with anti-Akt antibody and western blotting with anti-Nrf2 antibody. The results of Co-IP experiments confirmed the interaction between Akt and Nrf2 in PDLSCs, which was more obvious when using t-BHQ, the Nrf2 activator. These data further demonstrated that PI3K/Akt signaling pathway was involved in the Nrf2 activation in PDLSCs during the cyclic mechanical stress-stimulated osteogenic differentiation.

### The Identification and Validation of SOD2 Expression in PDLSCs During the Cyclic Mechanical Stress-Stimulated Osteogenic Differentiation

**FIGURE 4:**
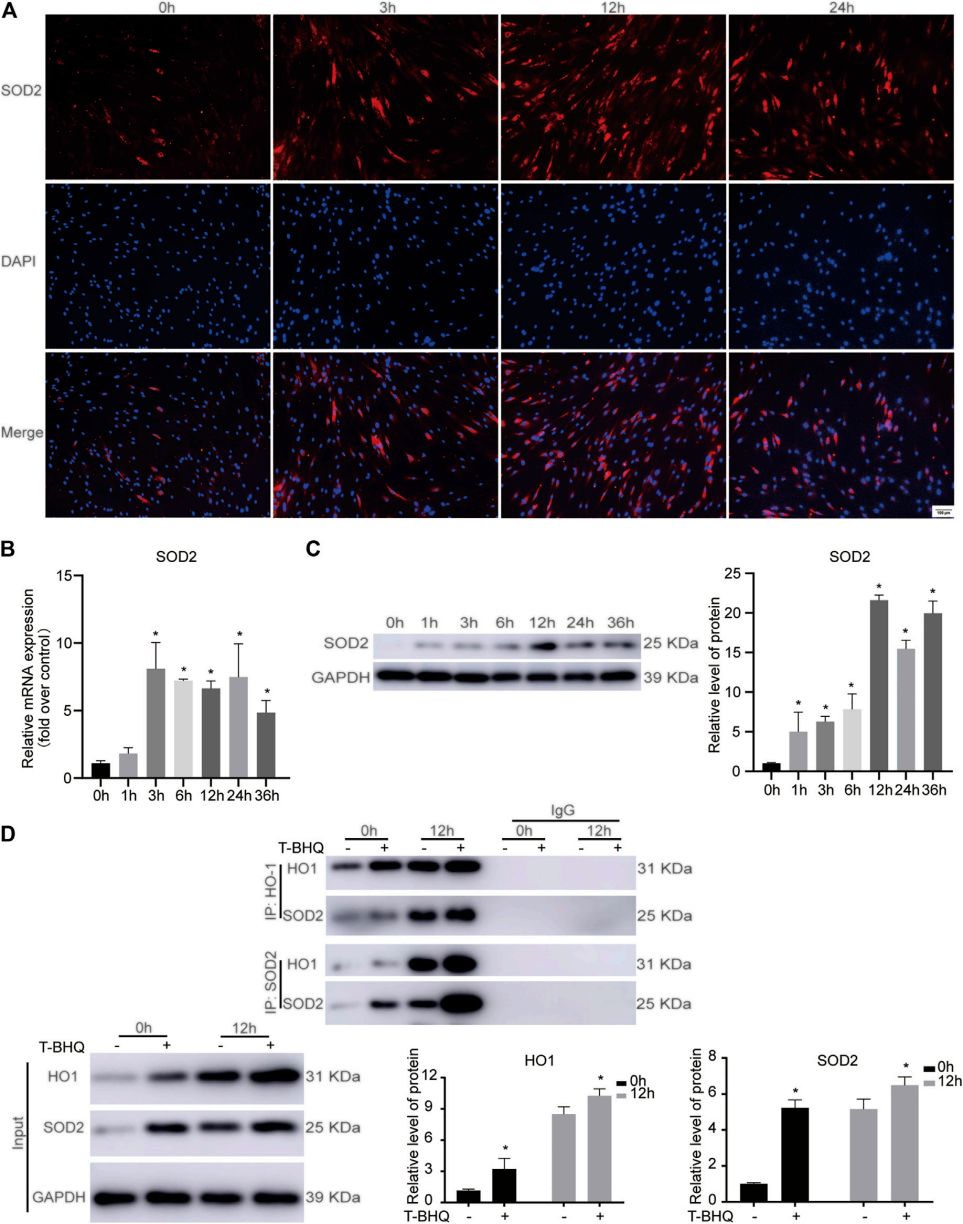
The protein-protein interaction between HO1 and SOD2 in PDLSCs under cyclic mechanical stress. **(A)** Immunofluorescence staining of SOD2 during cyclic mechanical stress in PDLSCs. The scale bars, 100 µm. **(B)** Cyclic mechanical stress up-regulated the SOD2 mRNA expression in PDLSCs, according to the results of RT-PCR. **(C)** Cyclic mechanical stress promoted the SOD2 protein expression in PDLSCs, according to the results of western blotting. **(D)** Co-IP experiments showed the protein-protein interaction between HO1 and SOD2 in PDLSCs as immunoprecipitation with anti-HO1 antibody and anti-SOD2 antibody to enrich the protein complex, respectively.

Among the differentially expressed proteins from TMT-based LC-MS/MS analysis, the potential protein-protein interaction between HO1 and SOD2, the downstream antioxidants of Nrf2, might be related to cyclic mechanical stress-induced osteoblast differentiation (Supplementary Table S4). Our previous research showed that cyclic mechanical stress promoted the expression of Nrf2 and its downstream antioxidant HO1. In the following study, we evaluated the SOD2 expression in PDLSCs. The data showed that the mRNA and protein expression of SOD2 were up-regulated during cyclic mechanical stress (Figures 4A–C and Supplementary Table S5). The data implied that the potential interaction between HO1 and SOD2 in PDLSCs might be associated with cyclic mechanical stress-stimulated osteoblast differentiation.

### The Protein-Protein Interaction Between HO1 and SOD2 in PDLSCs During the Cyclic Mechanical Stress-Stimulated Osteogenic Differentiation

To further confirm the involvement of HO1 and SOD2 interaction in PDLSCs during the cyclic mechanical stress, Co-IP experiments were carried out. Immunoprecipitation experiments were performed with anti-HO1 antibody to enrich the protein complex containing HO1, followed by western blotting with anti-SOD2 antibody. The data showed that SOD2 was present in the complex containing HO1. The similar result was observed when immunoprecipitation with anti-SOD2 antibody and western blotting with anti-HO1 antibody. According to the results of Co-IP experiments (Figure 4D), we confirmed that the interaction between HO1 and SOD2 was associated with the cyclic mechanical stress-stimulated osteogenic differentiation.

### The Effect of Nrf2 Activation on Osteogenic Relative Markers in Orthodontic Rats

**FIGURE 5:**
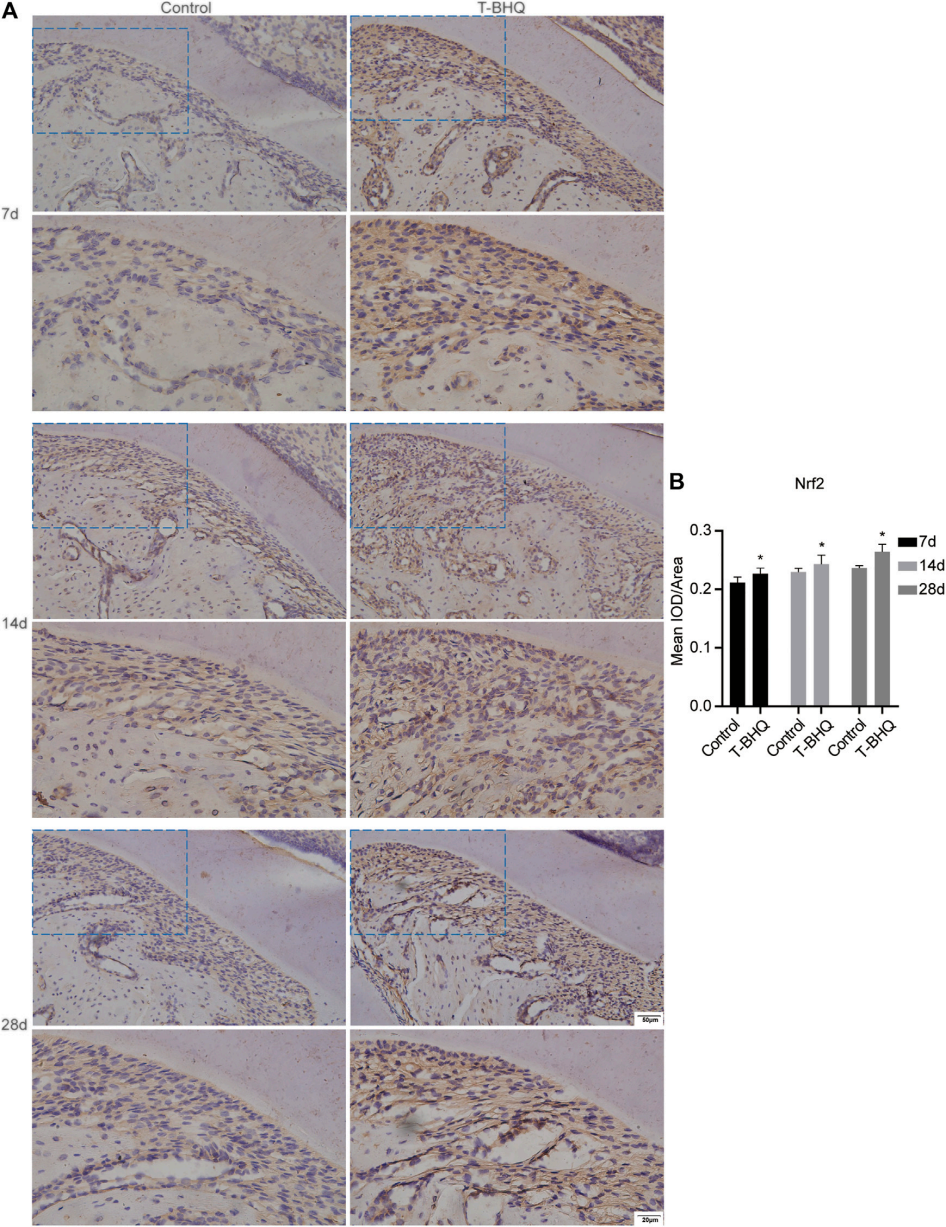
The increased expression of Nrf2 in the PDL at the tension side in orthodontic rats with t-BHQ treatment. **(A)** T-BHQ, the Nrf2 activator, could promote Nrf2 expression in the PDL at the tension side in orthodontic rats through IHC staining. The scale bars, 20 and 50 µm. **(B)** The mean integrated optical density (IOD)/Area of Nrf2 was quantified through Image-Pro Plus 6.0 software. N = 3 specimens in each group.

**FIGURE 6:**
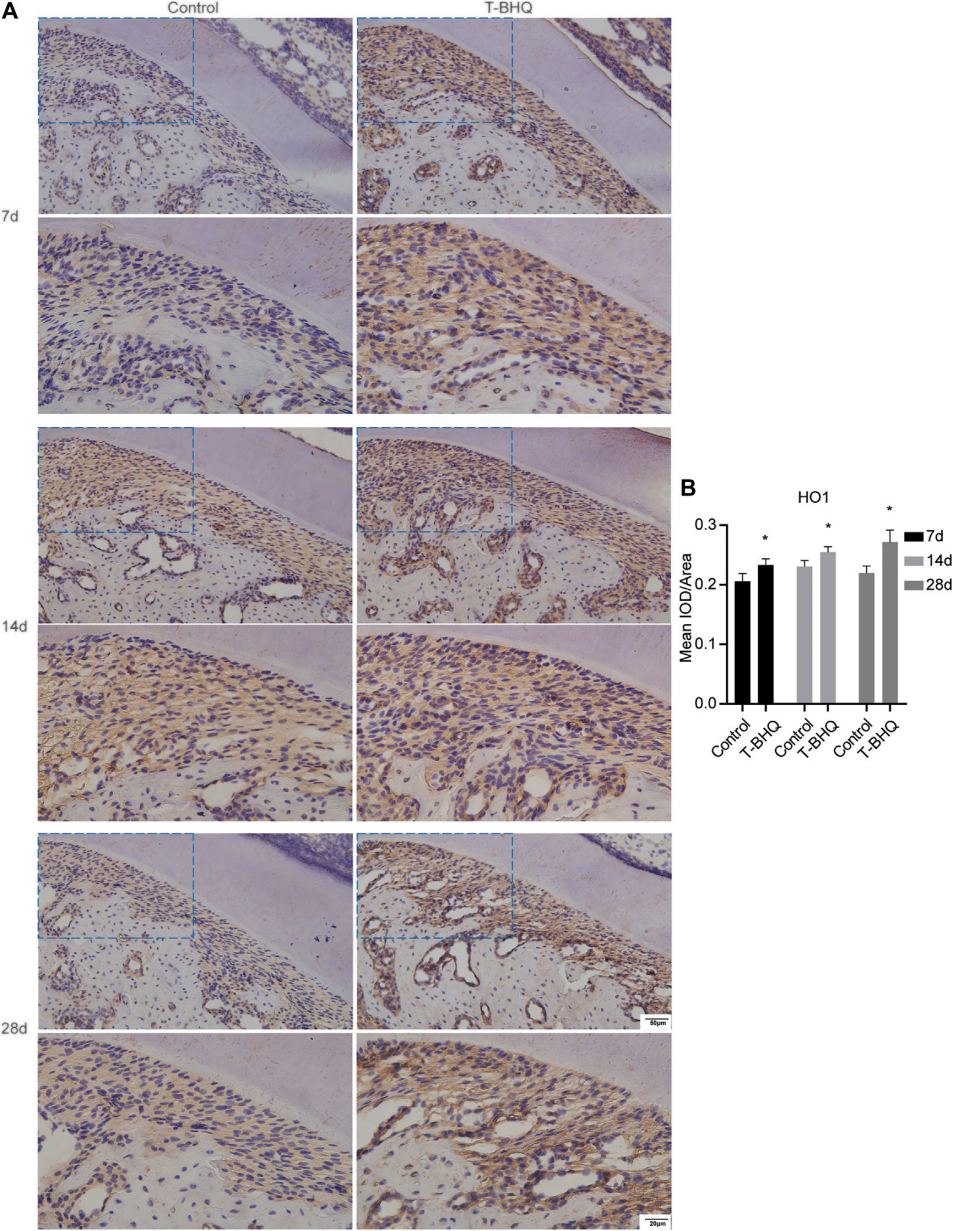
The increased expression of HO1 in the PDL at the tension side in orthodontic rats with t-BHQ treatment. **(A)** T-BHQ could promote the expression level of HO1, the downstream antioxidant of Nrf2, in the PDL at the tension side in orthodontic rats through IHC staining. The scale bars, 20 and 50 µm. **(B)** The mean IOD/Area of HO1 was quantified through Image-Pro Plus 6.0 software. N = 3 specimens in each group.

**FIGURE 7:**
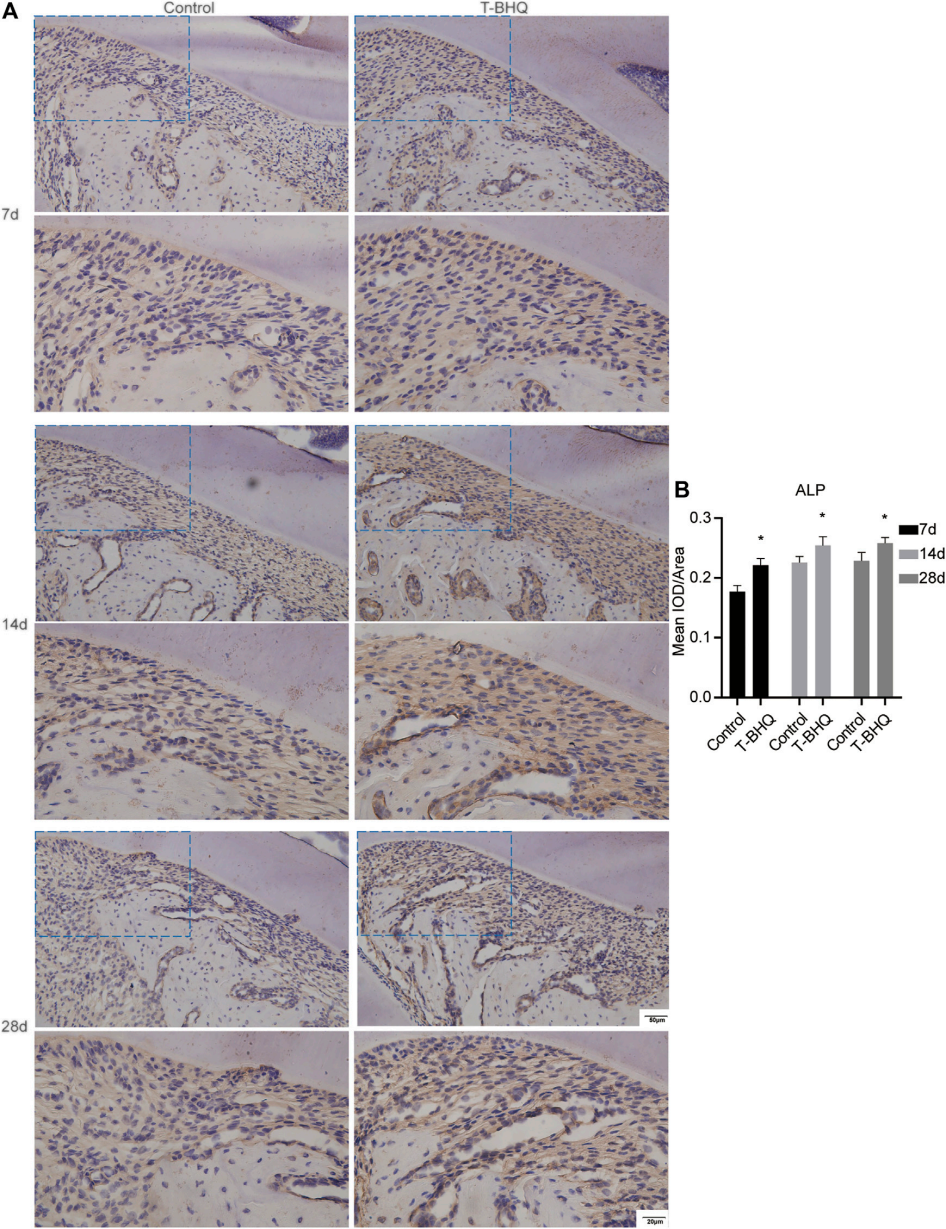
The increased expression of ALP in the PDL at the tension side in orthodontic rats with t-BHQ treatment. **(A)** The expression of ALP was enhanced with t-BHQ treatment in the PDL at the tension side in orthodontic rats through IHC staining. The scale bars, 20 and 50 µm. **(B)** The mean IOD/Area of ALP was quantified through Image-Pro Plus 6.0 software. N = 3 specimens in each group.

**FIGURE 8:**
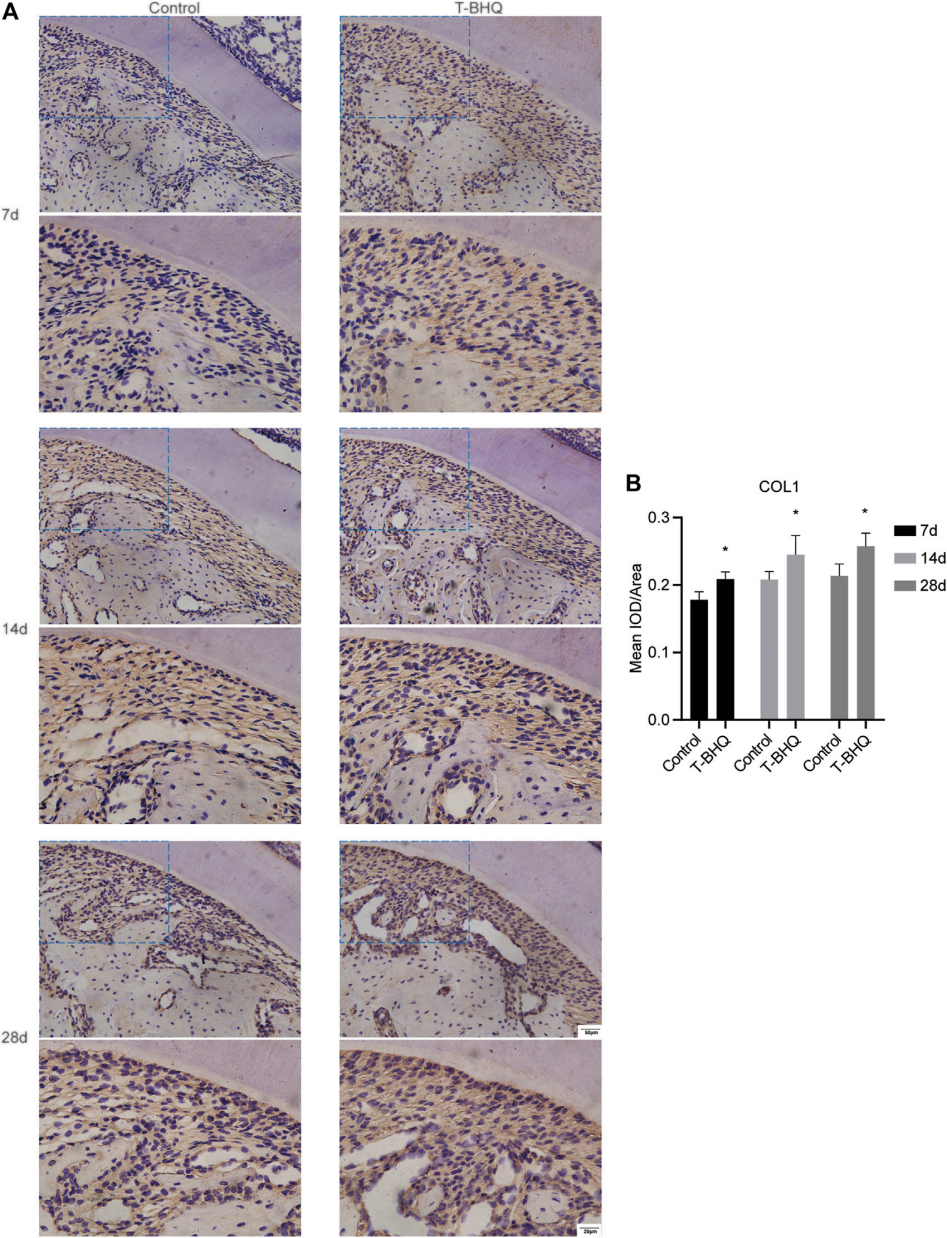
The increased expression of COL1 in the PDL at the tension side in orthodontic rats with t-BHQ treatment. **(A)** The expression of COL1 was up-regulated with t-BHQ treatment in the PDL at the tension side in orthodontic rats through IHC staining. The scale bars, 20 and 50 µm. **(B)** The mean IOD/Area of COL1 was quantified through Image-Pro Plus 6.0 software. N = 3 specimens in each group.

Our previous research revealed that Nrf2 had the positive effect on cyclic mechanical stress-stimulated osteoblast differentiation. To verify that Nrf2 might be a promising therapeutic target in orthodontics, we applied t-BHQ, the Nrf2 activator and the FDA-approved food additive, to orthodontic rats. And we detected the expression levels of osteogenic relative markers in PDL through IHC staining. T-BHQ could promote the expression of Nrf2 and its downstream antioxidant HO1 in PDL in orthodontic rats at the tension side through IHC staining (Figures 5, 6 and Supplementary Table S6). And the expression levels of ALP and COL1, the osteogenic relative markers, in orthodontic rats were up-regulated with t-BHQ treatment (Figures 7, 8 and Supplementary Table S6). These results indicated that Nrf2 might be a potential and promising target for therapeutic intervention to have positive effects on alveolar bone remodeling in orthodontics.

## Discussion

Our previous research revealed that Nrf2 played the beneficial role in cyclic mechanical stress-stimulated osteogenic differentiation in PDLSCs (Xi et al., 2021). Our current study was performed to explore the potential molecular mechanisms of Nrf2 in this process via TMT-based LC-MS/MS analysis. The mechanisms of Nrf2 underlying cyclic mechanical stress-stimulated osteogenic differentiation were associated with PI3K/Akt signaling pathway and HO1-SOD2 interaction in PDLSCs. And we applied t-BHQ, the Nrf2 activator, to the orthodontic rats and observed the increased expression levels of osteogenic relative markers, including ALP and COL1 by IHC staining. Nrf2 might be a promising therapeutic target to have favorable effects on alveolar bone remodeling in orthodontics.

PI3K/Akt signaling, the pivotal signaling pathway in cells, is related to various cell functions and processes, including cell proliferation, survival, metabolism and so forth (Mishra et al., 2021). Nrf2, the master regulator in antioxidant defense, is implicated in modulating the expression and activity of antioxidant components, including HO1 and NQO1 (Yu and Xiao, 2021). Previous researches showed that PI3K/Akt pathway was related to the activation and translocation of Nrf2 to regulate the synthesis of some protective protein including HO1 (Rai et al., 2019). Many studies demonstrated that PI3K/Akt might participate in osteogenic differentiation in PDLSCs. Metformin could reduce the level of ROS stimulated by H_2_O_2_ and promote osteogenic differentiation in PDLSCs, which was associated with PI3K/Akt/Nrf2 signaling pathway (Jia et al., 2020). Curcumin could have the beneficial effect on osteogenic differentiation in PDLSCs, and this was linked to the activation of PI3K/Akt/Nrf2 signaling pathway (Xiong et al., 2020).

According to the KEGG pathway analysis in our study, PI3K/Akt signaling pathway might be related to the Nrf2 activation in PDLSCs during the cyclic mechanical stress-stimulated osteogenic differentiation. And we applied LY294002, the PI3K inhibitor, to interfere with the Akt signaling in PDLSCs and observed the down-regulated expression of Nrf2 and the decreased cyclic mechanical stress-stimulated osteogenic differentiation. According to the STRING database and the results of Co-IP experiments, we confirmed the protein-protein interaction between Akt and Nrf2 in PDLSCs under cyclic mechanical stress, and this was more obvious when using t-BHQ, the Nrf2 activator. The abovementioned results revealed that PI3K/Akt pathway was involved in modulating the Nrf2 expression in cyclic mechanical stress-stimulated osteoblast differentiation of PDLSCs, which was agreement with the previous studies (Jia et al., 2020; Xiong et al., 2020). The protein-protein interaction between Akt and Nrf2 may affect the structure and stability of Nrf2. This requires further investigation in the future.

Among the differentially expressed proteins from TMT-based LC-MS/MS analysis, the potential protein-protein interaction between HO1 and SOD2 in PDLSCs might be implicated in cyclic mechanical stress-stimulated osteoblast differentiation. Our earlier research showed that cyclic mechanical stress enhanced the activation of Nrf2 and its downstream antioxidant HO1. And t-BHQ reinforced the expression levels of Nrf2 and HO1 as well as improved cyclic mechanical stress-induced osteoblast differentiation of PDLSCs (Xi et al., 2021).

HO1 possesses powerfully cytoprotective and antioxidant properties through the enzymatic degradation of heme (Consoli et al., 2021; Nitti et al., 2021). Some studies showed that HO1 had the crucial effect on regulating osteogenic differentiation. Panax ginseng fruit extract (PGFE) induced the expression levels of Nrf2 and HO1, and recovered the osteoblast differentiation restrained by *Porphyromonas gingivalis* lipopolysaccharide (PG-LPS) in the human periodontal ligament cells, while tin protoporphyrin IX (SnPP), the inhibitor of HO1, suppressed the osteoblast differentiation (Kim et al., 2020). Up-regulation of HO1 expression could improve the osteogenic differentiation in mesenchymal stem cells (MSCs) line C3H10T1/2 and promote ectopic bone formation in nude mice (Liu et al., 2018).

SOD2, an antioxidant enzyme, could catalyze superoxide radical anion (O_2_^•-^) into hydrogen peroxide (H_2_O_2_) in mitochondria (Kasai et al., 2020). And Nrf2 could induce the expression of SOD2 (Bellezza et al., 2018; Kasai et al., 2020). Our previous investigation indicated that cyclic mechanical stress reinforced the ROS level, including O_2_^•-^ and H_2_O_2_ in PDLSCs. In our current study, we observed the increased mRNA and the increased protein expression of SOD2 under cyclic mechanical stress in PDLSCs. And previous investigations revealed that SOD2 could be crucial for osteoblast differentiation. Melatonin restored the impaired osteogenic capacity of bone marrow mesenchymal stem cells (BMMSCs) stimulated by titanium (Ti) wear particles and improved intracellular antioxidant properties by increasing SIRT1 expression and up-regulating SOD2 activity (Zhang et al., 2020). Down-regulation of SOD2 expression induced by knocking down of SIRT3 was related to the reduced osteogenic differentiation, while SOD2 overexpression suppressed the reduction of osteogenic differentiation mediated by SIRT3 knockdown (Ding et al., 2017).

The abovementioned studies showed that HO1 and SOD2, as the important antioxidant enzymes and the downstream antioxidants of Nrf2, were associated with osteoblast differentiation. In order to confirm the potential protein-protein interaction between HO1 and SOD2, Co-IP experiments were carried out. The interaction between HO1 and SOD2 was observed in cyclic mechanical stress-stimulated osteoblast differentiation of PDLSCs. The interaction between HO1 and SOD2 might be related to the change of enzyme structure and activity and might affect the cyclic mechanical stress-stimulated osteoblast differentiation of PDLSCs. This is worthy of further study in the future.

Nrf2 played the beneficial role in cyclic mechanical stress-stimulated osteoblast differentiation in PDLSCs. This showed the possibility of t-BHQ, the Nrf2 activator, to be a potential and promising therapeutic target to improve the alveolar bone remodeling in orthodontics. T-BHQ, the food additive approved by FDA, was widely used in various animal models, such as traumatic brain injury (TBI) and hypertension (Bai et al., 2017; Chandran et al., 2018). To determine the positive effect of Nrf2 on osteogenic differentiation, we applied t-BHQ to the orthodontic rats. Our data showed that t-BHQ could up-regulate the Nrf2 expression and increase the expression levels of the osteogenesis markers (ALP and COL1) in orthodontic rats. Our data could be in line with previous researches, indicating that Nrf2 was crucial for osteoblast differentiation and bone formation (Ibáñez et al., 2014; Lippross et al., 2014; Wu et al., 2019; Zhu et al., 2020). Combined with our previous study, t-BHQ could play the positive role in BV/TV, Tb.Th, Tb.Sp as well as SMI, the microarchitectural parameters of trabecular bone in orthodontic rats (Xi et al., 2021). This further revealed that Nrf2 might be a promising and attractive target for therapeutic intervention in orthodontics.

The Akt-Nrf2 interaction might affect the structure and stability of Nrf2 and this might be associated with cyclic mechanical stress-stimulated osteoblast differentiation in PDLSCs. And the HO1-SOD2 interaction might be related to the change of enzyme structure and activity and this might affect cyclic mechanical stress-stimulated osteoblast differentiation in PDLSCs. These related molecular mechanisms require further investigation.

In conclusion, Nrf2 activation is involved in cyclic mechanical stress-stimulated osteogenic differentiation in PDLSCs via PI3K/Akt signaling and HO1-SOD2 interaction. T-BHQ, the Nrf2 activator, enhanced the expression levels of osteogenesis markers in orthodontic rats at the tension side. Nrf2 might be a potential and promising therapeutic target to improve the alveolar bone remodeling in orthodontic treatment.

## Data Availability

The original contributions presented in the study are included in the article/Supplementary Material, further inquiries can be directed to the corresponding author.

## References

[B1] An J. Li Y. Liu Z. Wang R. Zhang B. (2017). A Micro-CT Study of Microstructure Change of Alveolar Bone during Orthodontic Tooth Movement under Different Force Magnitudes in Rats. Exp. Ther. Med. 13, 1793–1798. 10.3892/etm.2017.4186 28565769 PMC5443305

[B2] Bai J. Yu X.-J. Liu K.-L. Wang F.-F. Jing G.-X. Li H.-B. (2017). Central Administration of Tert -butylhydroquinone Attenuates Hypertension via Regulating Nrf2 Signaling in the Hypothalamic Paraventricular Nucleus of Hypertensive Rats. Toxicol. Appl. Pharmacol. 333, 100–109. 10.1016/j.taap.2017.08.012 28842207

[B3] Bellezza I. Giambanco I. Minelli A. Donato R. (2018). Nrf2-Keap1 Signaling in Oxidative and Reductive Stress. Biochim. Biophys. Acta (BBA) - Mol. Cel Res. 1865, 721–733. 10.1016/j.bbamcr.2018.02.010 29499228

[B4] Chandran R. Kim T. Mehta S. L. Udho E. Chanana V. Cengiz P. (2018). A Combination Antioxidant Therapy to Inhibit NOX2 and Activate Nrf2 Decreases Secondary Brain Damage and Improves Functional Recovery after Traumatic Brain Injury. J. Cereb. Blood Flow. Metab. 38, 1818–1827. 10.1177/0271678X17738701 29083257 PMC6168911

[B5] Chen X. Hu C. Wang G. Li L. Kong X. Ding Y. (2013). Nuclear Factor-κB Modulates Osteogenesis of Periodontal Ligament Stem Cells through Competition with β-catenin Signaling in Inflammatory Microenvironments. Cell. Death Dis. 4, e510. 10.1038/cddis.2013.14 23449446 PMC3734811

[B6] Cheng Y. Liu M. Tang H. Chen B. Yang G. Zhao W. (2021). ITRAQ-Based Quantitative Proteomics Indicated Nrf2/OPTN-Mediated Mitophagy Inhibits NLRP3 Inflammasome Activation after Intracerebral Hemorrhage. Oxidative Med. Cell Longevity 2021, 1–26. 10.1155/2021/6630281 PMC7892225 33628368

[B7] Consoli V. Sorrenti V. Grosso S. Vanella L. (2021). Heme Oxygenase-1 Signaling and Redox Homeostasis in Physiopathological Conditions. Biomolecules 11, 589. 10.3390/biom11040589 33923744 PMC8072688

[B8] Dai X. Yan X. Wintergerst K. A. Cai L. Keller B. B. Tan Y. (2020). Nrf2: Redox and Metabolic Regulator of Stem Cell State and Function. Trends Mol. Med. 26, 185–200. 10.1016/j.molmed.2019.09.007 31679988

[B9] Ding Y. Yang H. Wang Y. Chen J. Ji Z. Sun H. (2017). Sirtuin 3 Is Required for Osteogenic Differentiation through Maintenance of PGC-1ɑ-SOD2-Mediated Regulation of Mitochondrial Function. Int. J. Biol. Sci. 13, 254–264. 10.7150/ijbs.17053 28255277 PMC5332879

[B10] Fão L. Mota S. I. Rego A. C. (2019). Shaping the Nrf2-ARE-Related Pathways in Alzheimer's and Parkinson's Diseases. Ageing Res. Rev. 54, 100942. 10.1016/j.arr.2019.100942 31415806

[B11] Francisqueti-Ferron F. V. Ferron A. J. T. Garcia J. L. Silva C. C. V. D. A. Costa M. R. Gregolin C. S. (2019). Basic Concepts on the Role of Nuclear Factor Erythroid-Derived 2-like 2 (Nrf2) in Age-Related Diseases. Ijms 20, 3208. 10.3390/ijms20133208 PMC6651020 31261912

[B12] Huang H. Yang R. Zhou Y.-h. (2018). Mechanobiology of Periodontal Ligament Stem Cells in Orthodontic Tooth Movement. Stem Cell Int. 2018, 1–7. 10.1155/2018/6531216 PMC6166363 30305820

[B13] Ibáñez L. Ferrándiz M. L. Brines R. Guede D. Cuadrado A. Alcaraz M. J. (2014). Effects of Nrf2 Deficiency on Bone Microarchitecture in an Experimental Model of Osteoporosis. Oxidative Med. Cell Longevity 2014, 1–9. 10.1155/2014/726590 PMC4121150 25120886

[B14] Jia L. Xiong Y. Zhang W. Ma X. Xu X. (2020). Metformin Promotes Osteogenic Differentiation and Protects against Oxidative Stress-Induced Damage in Periodontal Ligament Stem Cells via Activation of the Akt/Nrf2 Signaling Pathway. Exp. Cel Res. 386, 111717. 10.1016/j.yexcr.2019.111717 31715142

[B15] Kasai S. Shimizu S. Tatara Y. Mimura J. Itoh K. (2020). Regulation of Nrf2 by Mitochondrial Reactive Oxygen Species in Physiology and Pathology. Biomolecules 10, 320. 10.3390/biom10020320 PMC7072240 32079324

[B16] Kim E.-N. Kim T.-Y. Park E. K. Kim J.-Y. Jeong G.-S. (2020). Panax Ginseng Fruit Has Anti-Inflammatory Effect and Induces Osteogenic Differentiation by Regulating Nrf2/HO-1 Signaling Pathway in *In Vitro* and *In Vivo* Models of Periodontitis. Antioxidants 9, 1221. 10.3390/antiox9121221 PMC7761716 33287198

[B17] Kook S.-H. Lim S.-S. Cho E.-S. Lee Y.-H. Han S.-K. Lee K.-Y. (2014). COMP-angiopoietin 1 Increases Proliferation, Differentiation, and Migration of Stem-like Cells through Tie-2-Mediated Activation of P38 MAPK and PI3K/Akt Signal Transduction Pathways. Biochem. Biophysical Res. Commun. 455, 371–377. 10.1016/j.bbrc.2014.11.025 25446117

[B18] Lazaro I. Lopez-Sanz L. Bernal S. Oguiza A. Recio C. Melgar A. (2018). Nrf2 Activation Provides Atheroprotection in Diabetic Mice through Concerted Upregulation of Antioxidant, Anti-inflammatory, and Autophagy Mechanisms. Front. Pharmacol. 9, 819. 10.3389/fphar.2018.00819 30108504 PMC6080546

[B19] Lippross S. Beckmann R. Streubesand N. Ayub F. Tohidnezhad M. Campbell G. (2014). Nrf2 Deficiency Impairs Fracture Healing in Mice. Calcif. Tissue Int. 95, 349–361. 10.1007/s00223-014-9900-5 25096517

[B20] Liu X. Ji C. Xu L. Yu T. Dong C. Luo J. (2018). Hmox1 Promotes Osteogenic Differentiation at the Expense of Reduced Adipogenic Differentiation Induced by BMP9 in C3H10T1/2 Cells. J. Cel. Biochem. 119, 5503–5516. 10.1002/jcb.26714 29377252

[B21] Mishra R. Patel H. Alanazi S. Kilroy M. K. Garrett J. T. (2021). PI3K Inhibitors in Cancer: Clinical Implications and Adverse Effects. Int J Mol Sci 22, 3464. 10.3390/ijms22073464 33801659 PMC8037248

[B22] Motoji H. To M. Hidaka K. Matsuo M. (2020). Vitamin C and Eggshell Membrane Facilitate Orthodontic Tooth Movement and Induce Histological Changes in the Periodontal Tissue. J. Oral Biosciences 62, 80–87. 10.1016/j.job.2020.01.006 32032751

[B23] Nitti M. Ivaldo C. Traverso N. Furfaro A. L. (2021). Clinical Significance of Heme Oxygenase 1 in Tumor Progression. Antioxidants 10, 789. 10.3390/antiox10050789 34067625 PMC8155918

[B24] Rafiuddin S. Yg P. K. Biswas S. Prabhu S. S. Bm C. Mp R. (2015). Iatrogenic Damage to the Periodontium Caused by Orthodontic Treatment Procedures: An Overview. Todentj 9, 228–234. 10.2174/1874210601509010228 PMC4541303 26312093

[B25] Rai S. N. Dilnashin H. Birla H. Singh S. S. Zahra W. Rathore A. S. (2019). The Role of PI3K/Akt and ERK in Neurodegenerative Disorders. Neurotox. Res. 35, 775–795. 10.1007/s12640-019-0003-y 30707354

[B26] Seo B.-M. Miura M. Gronthos S. Mark Bartold P. Batouli S. Brahim J. (2004). Investigation of Multipotent Postnatal Stem Cells from Human Periodontal Ligament. The Lancet 364, 149–155. 10.1016/S0140-6736(04)16627-0 15246727

[B27] Sun Y.-X. Xu A.-H. Yang Y. Li J. (2015). Role of Nrf2 in Bone Metabolism. J. Biomed. Sci. 22, 101. 10.1186/s12929-015-0212-5 26511009 PMC4625735

[B28] Suzuki S. S. Garcez A. S. Suzuki H. Ervolino E. Moon W. Ribeiro M. S. (2016). Low-level Laser Therapy Stimulates Bone Metabolism and Inhibits Root Resorption during Tooth Movement in a Rodent Model. J. Biophoton 9, 1222–1235. 10.1002/jbio.201600016 27647761

[B29] Wu K. Gong Z. Zou L. Ye H. Wang C. Liu Y. (2019). Sargassum Integerrimum Inhibits Oestrogen Deficiency and Hyperlipidaemia-Induced Bone Loss by Upregulating Nuclear Factor (Erythroid-derived 2)-like 2 in Female Rats. J. Orthopaedic Translation 19, 106–117. 10.1016/j.jot.2019.03.002 PMC6896726 31844618

[B30] Xi X. Zhao Y. Liu H. Li Z. Chen S. Liu D. (2021). Nrf2 Activation Is Involved in Osteogenic Differentiation of Periodontal Ligament Stem Cells under Cyclic Mechanical Stretch. Exp. Cel Res. 403, 112598. 10.1016/j.yexcr.2021.112598 33865812

[B31] Xiong Y. Zhao B. Zhang W. Jia L. Zhang Y. Xu X. (2020). Curcumin Promotes Osteogenic Differentiation of Periodontal Ligament Stem Cells through the PI3K/AKT/Nrf2 Signaling Pathway. Iran J. Basic Med. SciJ. Basic Med. Sci. 23, 954–960. 10.22038/IJBMS.2020.44070.10351 PMC7395181 32774819

[B32] Yoon D. S. Choi Y. Lee J. W. (2016). Cellular Localization of NRF2 Determines the Self-Renewal and Osteogenic Differentiation Potential of Human MSCs via the P53-SIRT1 axis. Cel. Death Dis. 7. e2093. 10.1038/cddis.2016.3 PMC4849161 26866273

[B33] Yu C. Xiao J.-H. (2021). The Keap1-Nrf2 System: A Mediator between Oxidative Stress and Aging. Oxidative Med. Cell Longevity 2021, 1–16. 10.1155/2021/6635460 PMC8106771 34012501

[B34] Zhang H. Davies K. J. A. Forman H. J. (2015). Oxidative Stress Response and Nrf2 Signaling in Aging. Free Radic. Biol. Med. 88, 314–336. 10.1016/j.freeradbiomed.2015.05.036 26066302 PMC4628850

[B35] Zhang Y. Zhu X. Wang G. Chen L. Yang H. He F. (2020). Melatonin Rescues the Ti Particle-Impaired Osteogenic Potential of Bone Marrow Mesenchymal Stem Cells via the SIRT1/SOD2 Signaling Pathway. Calcif. Tissue Int. 107, 474–488. 10.1007/s00223-020-00741-z 32767062

[B36] Zhu C. Zhao Y. Wu X. Qiang C. Liu J. Shi J. (2020). The Therapeutic Role of Baicalein in Combating Experimental Periodontitis with Diabetes via Nrf2 Antioxidant Signaling Pathway. J. Periodont Res. 55, 381–391. 10.1111/jre.12722 31854466

